# Adiponectin Levels Are Associated with White Matter Lesions (WMLs) and Cognitive Impairment

**DOI:** 10.1155/2022/9943250

**Published:** 2022-01-18

**Authors:** Hui Quan, Tongya Yu, Yingying Lin, Jie Pan, Bingjie Mao, Xuan Wang, Junchao Xie, Xueyuan Liu, Yanxin Zhao

**Affiliations:** Shanghai Tenth People's Hospital of Tongji University, Department of Internal Neurology, Middle Yanchang Rd. 301#, Zhabei District, Shanghai, China 200072

## Abstract

**Method:**

In the present study, 126 patients, 90 cases in the WML group and 36 cases in the control group, were analyzed to explore the relationship between adiponectin and WMLs. All patients underwent an MRI scan to assess whether white matter lesions happened. And the serum levels of adiponectin were detected by ELISA.

**Results:**

In this study, according to Fazekas criteria, WMLs were divided into different severity groups. With the increase of WML score, the level of adiponectin decreased, and linear correlation analysis shows that adiponectin is negatively correlated with the severity of white matter lesions (*p* < 0.001). And adiponectin level was significantly positively correlated with MoCA score (*p* < 0.05). Moreover, adiponectin in the WMLs combined with the cognitive impairment group was significantly reduced (*p* < 0.01).

**Conclusion:**

The level of adiponectin is independently associated with WMLs and cognitive function, which suggests that adiponectin may be a protective factor for WMLs and cognitive function.

## 1. Introduction

White matter lesions (WMLs), also named leukoaraiosis (LA), were characterized mainly by hyperintensities on T2-weighted or FLAIR images, which were first put forward in 1987 [1]. WMLs were thought to be related to chronic cerebrovascular hypoperfusion, disorders of endothelial cells, and cerebral small vessel diseases, which include ischemic and hemorrhagic lesions [2]. Existing studies demonstrated that WMLs were closely associated with cognitive decline, proven by postmortem findings of dementia patients with WMLs [3–5].

Adiponectin is an adipose tissue-derived protein, mainly existing in the peripheral circulation, which plays diverse functions in physiological and pathological processes including anti-inflammation, antiatherosclerosis, and insulin sensitivity [6]. Besides, Kato et al. have found that lack of adiponectin may lead to thrombosis and platelet aggregation [7]. And in the animals that suffered vascular surgeries, adiponectin was also found upregulated, which demonstrates that adiponectin might join in the repairment of vascular injury. Adiponectin was also demonstrated ameliorating the inflammatory responses [8–10]. In recent years, with the deepening of the understanding of adiponectin, more and more studies have found that adiponectin also plays a role in the central nervous system. And the receptors of adiponectin are found expressed in various areas of the brain, through which adiponectin functions. In the central nervous system, adiponectin was found participating in several physiological and pathological processes including cognitive function [11–13], while other studies have come to the opposite conclusions [14].

In the present study, we aimed to analyze the relationship between adiponectin and white matter lesions and cognitive decline by comparing serum adiponectin levels in the control group and WML patients with cognitive impairment.

## 2. Patients and Methods

### 2.1. Participants

Patients who were hospitalized in the Department of Neurology at Shanghai 10th People's Hospital were screened for this study from May 2017 to December 2017. Participants were divided into two groups (WML group and control group) according to if WMLs were found in T2-weighted fluid-attenuated inversion recovery (T2W-FLAIR).

The inclusion criteria for participants were listed as the following: (a) patients aged over 40 years old; (b) punctate or patchy hyperintense signal zone exists in the periventricule or centrum semiovale were found in the phase of T2-weighted fluid-attenuated inversion recovery (T2W-FLAIR); (c) agree to accept blood sampling after informed consent. Patients who met any of the following criteria were excluded from the study: (a) patients diagnosed with multiple sclerosis, adrenoleukodystrophy, intoxicated cerebropathy, white matter lesions induced by self-immune diseases, or genetic diseases; (b) patients diagnosed with acute stroke or suffered episodes of stroke within the latest two weeks; (c) patients with confirmed diagnosis of cerebral occupying lesions, trauma, severe dementia, and psychiatric diseases; (d) patients diagnosed with malignant diseases or severe infections; (e) patients with incomplete data; (f) participants with contradictions to MRI examination.

### 2.2. Baseline Information Collection

Baseline data were collected including sex, age, height, body weight, smoking status, alcohol consumption, and medical history including hypertension, diabetes, coronary arterial disease, stroke, dyslipidemia, and inherited genetic diseases.

### 2.3. MRI Assessment

All participants underwent a multimodality brain MRI scanning (MAGNETOM Verio 3 T, Siemens, Berlin and Munich, Germany), including T1-weighted imaging (TR/TE: 450/8.9 ms), T2-weighted imaging (TR/TE: 5000/87 ms), and T2W-FLAIR (TR/TE: 8500/88 ms, reversal time: 2000 ms) and diffusion-weighted imaging (DWI).

The diagnosis and classification for WMLs were made according to Fazekas score and standards established by the Leukoaraiosis and Disability Study Group (LADIS) [14, 15].

For periventricular WMLs, we scored based on the sizes and numbers of lesions as the following: 0: no disease or lesion diameter < 3 mm; 1 point: linear lesions near the ventricle or cucullate lesions near the ventricular foot; 2 points: smooth ribbon lesions or larger cucullate lesions near the ventricle; 3 points: irregular lesions near the ventricle, or affect the deep white matter. For deep WML, 0: no lesion or diameter of lesion < 3 mm; 1 point: single lesion diameter > 3 mm, and <10 mm, diameter < 20 mm for grouped lesions; 2 points: the diameter of single lesion within the range of 10-20 mm, or lesion diameter ≥ 20 mm for grouped lesions with no obvious fusion between lesions; 3 points: single or fused lesion diameter ≥ 20 mm. Finally, the scores of PVWML and DWML are added together, and patients are divided into mild groups (1~2 points), moderate groups (3~4 points), and severe groups (5~6 points). In this study, there were 37 cases, 37 cases, and 22 cases in the mild group, moderate group, and severe group, respectively.

All the scores and gradings of MRI were assessed by a senior radiologist and reviewed by another radiologist. If these radiologists make a different judgment, a third radiologist will be consulted for final results.

### 2.4. Assessment of Cognitive Function

The Montreal Cognitive Assessment (MoCA) scales were used to assess cognitive function of participants. The final scores were adjusted according to education by +1 point if the participant received less than 12 years of education. The evaluation was conducted and recorded by a professional physician.

### 2.5. Venous Blood Collection and Measurement

Measurement of laboratory parameters: venous blood was taken at fasting time of early morning within 24 hours of admission. The laboratory parameters were recorded including complete blood counts, C-reactive protein, electrolytes, blood sugar, glycosylated hemoglobin, liver function (including aspartate transferase, glutamate transferase, total protein, and albumin), renal function (including creatinine, uric acid, and urea nitrogen), and blood lipids (including total cholesterol, triglycerides, low-density lipoprotein, and high-density lipoprotein).

Detection of adiponectin: 5 mL of peripheral blood (for serum extraction) from all subjects was drawn with EDTA tubes and stored in a -80°C low-temperature refrigerator for later measurement. Blood serum was separated by centrifugation (Eppendorf 5425 CT, USA). Adiponectin levels were detected using the ELISA Kit for human adiponectin (USCN Life Science Inc., Belgium). The relative concentration was determined in comparison with a predetermined standardized curve at the wavelength of 450 nm via the utilization of the Model 680 Microplate Reader according to the instructions (Bio-Rad, CA, USA).

### 2.6. Statistical Analysis

All the statistical analyses were carried out via SPSS 19.0 software (IBM, NY, USA). Normal distribution measurement data were expressed as *x* ± *s*, and nonnormal distribution measurement data were described by median using the rank-sum test. The student *t*-test analysis was used to compare the mean of the measurement data that conformed to the normal distribution, and the Mann-Whitney test or the Kolmogorov-Smirnov test was used for the statistics of the data without conformation to normality. Categorical data is expressed by composition ratio (%). The comparison of rates between groups was statistically analyzed by *χ*^2^ or Spearman rank correlation test. A *p* value less than 0.05 was considered statistically significant.

### 2.7. Ethical Statement

This study was approved by the Ethics Committee of Shanghai Tenth People's Hospital (No.: SHSY-IEC-4.0/17-20/01). All participants signed the informed consent. The study was performed according to Helsinki Declaration (as revised in 2013).

## 3. Results

### 3.1. Demographic and Clinical Characteristics

In the present study, 126 participants were enrolled, 96 participants in the WML group and 30 cases in the control group. Of 126 participants, 65 (51.59%) patients were male, and the age of participants varied from 53 to 85 years old. In the WML group, 50 (52.08%) participants were male; the mean age was 71.79 ± 8.78 years old. In the control group, 15 (50.00%) participants were male; the mean age was 67.37 ± 6.23 years old. The details are provided in Table 1.

In the comparison of gender, age, BMI, education years, smoking history, drinking history, and other baseline data, there were significant differences in age, BMI, and education years between the two groups that patients in the WML group were older, fatter, and had received shorter education time than participants in the control group (*p* < 0.05, *p* < 0.5, and *p* < 0.001, respectively) (Table 1). And comparing the medical history of stroke, hypertension, diabetes, coronary heart disease, and hyperlipidemia between the two groups, there was a significant difference in the history of hypertension that the proportion of patients with hypertension in the WML group was higher than that in the control group (78.10% vs. 36.70%, *p* < 0.001), and other medical histories of the two groups were similar (Table 1).

### 3.2. Laboratory Parameters at Admission

When comparing the blood pressure at admission of the control group and the WML group, no significant difference was observed both in systolic and diastolic blood pressures. And in the laboratory parameters, patients in the WML group had higher total cholesterol, HDL, and serum creatinine level but lower LDL level (*p* < 0.05, *p* < 0.05, *p* < 0.05, and *p* < 0.01, respectively), while other laboratory parameters were similar between the two groups. And in the evaluation of cognitive function, patients in the WML group had lower cognitive score than those in the control group, whether by using MMSE and MOCA scales (*p* < 0.001 and *p* < 0.001, respectively) (Table 2). And data showed that the adiponectin level in the WML group was significantly lower than the control group (*p* < 0.01) (Table 2).

### 3.3. Correlation between WMLs and Relevant Risk Factors

When participants were divided into 3 groups according to WML score: mild WMLs (1-2 points, *n* = 37), moderate WMLs (3-4 points, *n* = 37), and severe WMLs (5-6 points, *n* = 22), analysis of the association of adiponectin and the severity of WMLs showed that WML score was significantly correlated with adiponectin level (*p* < 0.001, Table 3). And patients with higher WML score had lower adiponectin level. When the risk factors of WMLs were analyzed, the results showed that there was a linear correlation between the score of WMLs with the levels of HDL besides adiponectin. WML score was positively correlated with HDL level that patients with higher WML score had higher HDL level, which suggests that HDL may be a risk factor for WMLs, while adiponectin may be a protective factor (Table 4).

### 3.4. Correlation of Levels of Adiponectin and Cognitive Function

We evaluated the relationship between cognitive score and adiponectin level via Spearman correlation analysis; the results showed that the adiponectin level was positively correlated with MoCA score (*p* < 0.05) that patients with higher adiponectin level tend to get higher MOCA score. When participants were divided into three groups according to whether they had WMLs and cognitive impairment, group 1: the control group, group 2: patients with WMLs but without cognitive defects, and group 3: patients with both WMLs and cognitive defects, comparison of adiponectin level between 3 groups showed that adiponectin level was reduced significantly in group 3 compared with group 1 (*p* < 0.05) that adiponectin level was lower in patients with white matter lesions and cognitive impairment compared with the control group.

## 4. Discussion

In this study, according to Fazekas criteria, white matter lesion (WML) was divided into different severity groups. With the increase of WML score, the level of adiponectin decreased, and linear correlation analysis shows that adiponectin level is negatively correlated with the severity of white matter lesions while positively correlated with MoCA score. And adiponectin in the WMLs combined with the cognitive impairment group was significantly reduced compared with the control group. Taken together, these results suggest that adiponectin may be a protective factor for WMLs and cognitive impairment.

White matter lesion (WML) is a common imaging type of cerebral small vascular disease which may induce cognitive impairment, gait disorder, urinary incontinence, depression, and other symptoms [15]. In the present study, patients with WMLs tend to have lower cognitive score compared with the control group which is consistent with published studies. In Lam et al.'s study, 5701 participants from 9 Asian cities were analyzed and results showed that WML was negatively associated with MMSE score in all groups [16]. And Wu et al. have enrolled 487 patients with ischemic stroke and followed up for 3 years. The results showed that white matter lesions were a risk factor for cognitive impairment after stroke [17]. And published studies showed that a variety of risk factors may promote the occurrence of WMLs, including age, blood pressure, blood sugar, blood lipids, high-sensitivity C-reactive protein, and homocysteine.

Adiponectin is an endogenous biologically active polypeptide secreted by adipocytes. Existing studies have shown that adiponectin has several effects such as antiatherosclerosis, improving endothelial function, improving vascular remodeling, reducing the inflammatory response of blood vessel walls, and increasing insulin sensitivity by binding to the corresponding receptors of target organs [18]. And adiponectin is reported involving in maintaining the integrity of brain function and plays a role in the pathophysiology of neurodegenerative diseases. In the present study, the data show that WML score was significantly correlated with adiponectin level and patients with higher WML score tend to have lower adiponectin level, which is consistent with published studies [19–21]. However, our data showed that the more severe the white matter lesions, the lower the adiponectin level which is contrary to the results of Youshi et al.'s study [19]. Their results believe that the severity of white matter lesions is positively correlated with the adiponectin level, and the more serious the white matter lesions, the higher the adiponectin level. The difference may be attributable to the different assessment methods used in each study. In Youshi et al.'s study, cranial CT and Blennow's visual rating scale were used to identify and evaluate white matter lesions, while we use cranial MRI and the Fezakas scoring scale, which scores PVWML and DWML separately and has an advantage of simple, accurate, easy to operate, and suitable for clinical projects [20]. Different evaluation methods may lead to different results that cranial MR scan is more sensitive to the identification of white matter lesions than cranial CT. In addition, the small sample size of both studies is an important reason for these differences. And Takahashi et al.'s findings [21] suggest that accumulation of adiponectin in the cerebral cortex may protect tissue injury by inhibiting inflammation under chronic cerebral hypoperfusion, which indicates that adiponectin may be beneficial to the brain.

Previous studies have shown that the level of adiponectin is also related to cognitive function which was consistent with the present study. Liu et al.'s study compared adiponectin levels in patients with type 2 diabetes mellitus with cognitive impairment and those with normal cognitive function, and data showed that adiponectin levels were significantly reduced in patients with cognitive impairment [22]. Cezaretto et al. analyzed the relationship between cognitive function and adiponectin level in 938 nondiabetic patients in the ELSA study, and the results showed that adiponectin level was independently associated with recall memory [23]. In addition to adults, adiponectin levels are also associated with children's cognitive function. Li et al.'s research shows that cord blood adiponectin is related to children's cognitive abilities [24]. In addition, the effect of adiponectin on cognitive function was also observed in animal experiments. In Bloemer et al.'s study, adiponectin knockout mice showed cognitive impairment and synaptic function changes [25]. And APN-transfected EPC have a beneficial effect on cognitive function in D-gal-induced aging rats. Besides, BBB dysfunction and angiogenesis were also improved, and neuroinflammation and apoptosis rate were reduced [26].

The present study contains some limitations. First of all, the sample size is small. Secondly, this study is an observational study, our results can only show that adiponectin level is related to the occurrence of white matter lesions and cognitive impairment, and how adiponectin affects the occurrence of white matter lesions needs further mechanism research. Thus, further randomized controlled trials with a much larger sample size are needed to reveal the mechanism of adiponectin to join in the formation of WMLs.

## 5. Conclusion

Adiponectin is correlated with the severity of WMLs and cognitive function, and adiponectin may be a protective factor for WMLs and cognitive impairment.

## Figures and Tables

**Table 1:** Baseline characteristics of participants.

	WML group	Control group	*F*/*Z*/*χ*^2^	*p* value
Demographic characteristics				
Age (years)	71.79 ± 8.78	67.37 ± 6.23	7.091	0.003^∗∗^
Male/*n* (%)	50 (52.10%)	15 (50.00%)	-0.199	0.843
BMI (kg/m^2^)	24.45 ± 3.15	23.09 ± 2.52	0.602	0.033^∗^
Educational years	8.88 ± 3.64	11.53 ± 3.26	-3.483	<0.001^∗∗∗^
Smoking (*n*/%)	37 (38.50%)	8 (26.70%)	1.404	0.236
Alcohol consumption	22 (22.90%)	6 (20.00%)	0.113	0.737
Medical history				
History of stroke (*n*/%)	28 (29.20%)	5 (16.70%)	1.848	0.174
Hypertension (*n*/%)	75 (78.10%)	11 (36.70%)	11.235	0.001∗∗
Diabetes (*n*/%)	28 (29.20%)	5 (16.70%)	1.848	0.174
Coronary artery disease (*n*/%)	24 (25.00%)	7 (23.30%)	0.034	0.853
Hyperlipidemia (*n*/%)	39 (40.60%)	9 (30.00%)	1.094	0.296

BMI: body mass index, measured by body height/body weight^2^. ^∗^*p* < 0.05; ^∗∗^*p* < 0.01; ^∗∗∗^*p* < 0.001.

**Table 2:** Baseline parameters of participants at admission.

	WML group	Control group	*F*/*Z*/*χ*^2^	*p* value
Blood pressure at admission				
Systolic pressure (mmHg)	145.55 ± 20.51	136.20 ± 27.94	-1.537	0.124
Diastolic pressure (mmHg)	79.78 ± 9.99	80.93 ± 10.49	-0.485	0.628
Laboratory parameters				
C-reactive protein (mg/L)	4.72 ± 9.46	2.65 ± 1.39	-1.653	0.098
White blood cell counts (10∗9/L)	6.24 ± 1.53	5.98 ± 1.34	0.862	0.387
Hemoglobin (g/L)	129.79 ± 18.97	133.77 ± 16.60	-0.808	0.419
Platelet counts (10∗9/L)	194.53 ± 43.17	184.97 ± 35.15	1.41	0.272
Glucose (mmol/L)	5.72 ± 1.81	5.65 ± 1.43	-0.788	0.431
HbA1c (%)	6.26 ± 1.41	6.18 ± 1.24	-0.046	0.963
Total cholesterol (mmol/L)	4.64 ± 1.01	4.15 ± 1.13	0.492	0.034
Total triglyceride (mmol/L)	1.62 ± 1.20	1.52 ± 0.88	-0.561	0.575
HDL (mmol/L)	1.30 ± 0.38	1.12 ± 0.34	-2.53	0.011^∗^
LDL (mmol/L)	2.63 ± 1.70	3.01 ± 0.90	-2.66	0.008^∗∗^
Hcy (*μ*mol/L)	11.14 ± 8.10	856 ± 2.23	-1.58	0.114
Serum creatinine (*μ*mol/L)	78.75 ± 24.72	70.09 ± 20.15	-2.074	0.038^∗^
Cognitive function assessment				
MMSE	25.58 ± 4.19	29.00 ± 1.20	-4.762	<0.001^∗∗∗^
MoCA	22.58 ± 5.01	28.17 ± 2.00	-6.413	<0.001^∗∗∗^
Adiponectin (*μ*g/L)				
Adiponectin (*μ*g/L)	1051.57 ± 142.30	1140.82 ± 129.92	1.038	0.003^∗∗^

HbA1c: hemoglobin A1c; HDL: high-density lipoprotein; LDL: low-density lipoprotein; IMT: intima-media thickness. ^∗^*p* < 0.05; ^∗∗^*p* < 0.01; ^∗∗∗^*p* < 0.001.

**Table 3:** The levels of adiponectin in the groups divided by severity of the WML—Kolmogorov-Smirnov test.

	*n*	Adiponectin
Control group	30	1140.82 ± 129.92
Group with mild WML	37	1148.67 ± 108.40
Group with moderate WML	37	1039.30 ± 136.52
Group with severe WML	22	923.88 ± 105.90
Correlation coefficient	—	0.505
*p* value	—	<0.001^∗∗∗^

^∗∗∗^ *p* < 0.001.

**Table 4:** The association of WMLs and relevant risk factors—multiple linear regression analysis.

Variables	Regression coefficient	OR value	Wald	*p* value	95% CI	
					Lower	Upper
Age	0.038	0.032	1.435	0.231	0.976	1.106
Gender	0.424	0.562	0.570	0.450	0.508	4.599
TC	-0.508	0.329	2.385	0.123	0.316	1.147
HDL	2.524	1.026	6.048	0.014^∗^	1.669	93.271
LDL	0.130	0.269	0.233	0.630	0.672	1.931
Creatine	0.012	0.014	0.014	0.388	0.985	1.039
Adiponectin	-0.004	1.002	5.458	0.019^∗^	0.992	0.999

^∗^ *p* < 0.05.

## Data Availability

Data are available upon request.
